# Impaired Autophagy and Defective T Cell Homeostasis in Mice with T Cell-Specific Deletion of Receptor for Activated C Kinase 1

**DOI:** 10.3389/fimmu.2017.00575

**Published:** 2017-05-18

**Authors:** Guihua Qiu, Jian Liu, Qianqian Cheng, Qingyang Wang, Zhaofei Jing, Yujun Pei, Min Zhao, Jing Wang, Jessie Yanxiang Guo, Jiyan Zhang

**Affiliations:** ^1^Department of Molecular Immunology, Institute of Basic Medical Sciences, Beijing, China; ^2^Graduate School, Guangxi Medical University, Nanning, China; ^3^Division of Medical Oncology, Rutgers Cancer Institute of New Jersey, RBHS-Robert Wood Johnson Medical School, New Brunswick, NJ, USA

**Keywords:** receptor for activated C kinase 1, T cells, autophagy, apoptosis, acute liver injury

## Abstract

Autophagy plays a central role in maintaining T cell homeostasis. Our previous study has shown that hepatocyte-specific deficiency of receptor for activated C kinase 1 (RACK1) leads to lipid accumulation in the liver, accompanied by impaired autophagy, but its *in vivo* role in T cells remains unclear. Here, we report that mice with T cell-specific deletion of RACK1 exhibit normal intrathymic development of conventional T cells and regulatory T (Treg) cells but reduced numbers of peripheral CD4^+^ and CD8^+^ T cells. Such defects are cell intrinsic with impaired mitochondrial clearance, increased sensitivity to cell death, and decreased proliferation that could be explained by impaired autophagy. Furthermore, RACK1 is essential for invariant natural T cell development. *In vivo*, T cell-specific loss of RACK1 dampens concanavalin A-induced acute liver injury. Our data suggest that RACK1 is a key regulator of T cell homeostasis.

## Introduction

T cells are crucial controllers of inflammation and immunity against pathogens and incipient tumors. Autophagy plays a central role in maintaining T cell homeostasis (1–9). As an evolutionarily conserved eukaryotic process, autophagy degrades long-lived proteins and other cytoplasmic contents such as aggregated proteins, damaged/excess organelles, and lipids through lysosomes (10–12). Although autophagy usually occurs in response to starvation and other stresses, this process also happens under nutrient-rich condition (basal or constitutive autophagy). T cell-specific deletion of autophagy-related genes Atg3, Atg5, Atg7, Atg16l1, Vps34, or Beclin 1 leads to decreased frequencies and numbers of peripheral CD4^+^ and CD8^+^ T cells (1–9). The clearance of damaged/excess organelles, especially mitochondria, is suggested to be the key mechanism by which autophagy promotes the survival of peripheral T lymphocytes (2–4, 6). Furthermore, previous results have indicated that Atg3-, Atg5-, Atg7-, or Vps34-deficient T cells cannot proliferate efficiently (2–4, 8, 9).

WD40 repeats are ~40 amino acid motifs, usually ending with a Trp-Asp (W-D) dipeptide (13). WD40-repeat proteins, a large family highly conserved in all eukaryotes, are implicated in a variety of functions, including cell cycle control and apoptosis (13, 14). Nevertheless, the *in vivo* functions of WD40 repeats have been studied less intensely than other common domains such as the kinase, PDZ, or SH3 domains (13, 14). The essential role of WD40-repeat-only proteins in postnatal mammalian physiology has only been disclosed recently (15). Receptor for activated C kinase 1 (RACK1; official gene name *Gnb2l1*) was originally identified on the basis of its ability to anchor activated form of protein kinase C (PKC). As a WD40-repeat-only protein, it has been recognized as an adaptor involved in multiple intracellular signaling pathways. We have shown that RACK1 promotes the autophagy-initiation complex formation. Consequently, RACK1 deficiency in hepatocytes leads to lipid accumulation in the liver (15). As for T cell biology, the possible involvement of RACK1 in T cell survival has been suggested (16). Despite that, the *in vivo* role of RACK1 in T cells remains unclear. In this work, we generated mice with specific deletion of RACK1 in T cells and identified RACK1 as a new regulator of T cell homeostasis.

## Materials and Methods

### Mice

Mice homozygous for a *Gnb2l1* conditional allele (*Gnb2l1*^F/F^) were previously described and were all backcrossed to the C57BL/6 background (15). Mice expressing a transgene encoding Cre recombinase driven by the lymphocyte-specific protein tyrosine kinase proximal promoter (*Lck-Cre* mice) (17) and under the control of CD4 promoter (*CD4-Cre* mice) (18, 19) were gifts from Dr. Hua Han (The Fourth Military Medical University, Xi’an, China) and Dr. Chen Dong (Tsinghua University, Beijing, China), respectively. Specific inactivation of RACK1 in T cells was achieved by crossing *Gnb2l1*^F/F^ mice with *Lck*-*Cre* mice or *CD4-Cre* mice. *Gnb2l1*^F/F; lck-Cre^ mice were kept in the animal facility of Institute of Biotechnology (20 Dongdajie, Beijing, China), whereas *Gnb2l1*^F/F; CD4-Cre^ mice were kept in the animal facility of Institute of Basic Medical Sciences (27 Taiping Road, Beijing, China). All mice were maintained under specific pathogen-free conditions and animals were handled in accordance with institutional guidelines. Genotyping was performed as described previously (15).

### Flow Cytometry Analysis of Cell Surface Markers and Intracellular Cytokines/Foxp3

Fluorescence-labeled antibodies against CD3, CD4, CD8, CD25, CD44, CD62L, CD69, CD19, CD45.1, CD45.2, NK1.1, IFN-γ, and TNF-α were purchased from eBioscience. Fluorescence-conjugated antibody against Foxp3 was from BioLegend. α-GalCer-loaded recombinant murine CD1d tetramers (E001-2A-G) were purchased from ProImmune. Cells from thymi, spleens (SPLs), and mesenteric lymph nodes (mLNs) were depleted of erythrocytes by hypotonic lysis. The cells were washed with FACS washing buffer (2% FBS, 0.1% NaN_3_ in PBS) twice and were then incubated with fluorescence-conjugated antibodies against cell surface molecules for 30 min on ice in the presence of 2.4G2 mAb to block FcγR binding. Isotype antibodies were included as negative controls. For Foxp3 and intracellular cytokine staining, single-cell suspensions were stained with fluorescence-conjugated antibodies against cell surface markers, fixed and permeabilized using a fixation/permeabilization kit (eBioscience), and stained with fluorescence-conjugated specific antibodies against Foxp3 and cytokines in accordance with the manufacturer’s instructions. Flow cytometry was performed using a Becton Dickinson FACSCalibur machine.

### Antibodies and Immunoblotting (IB)

Antibodies against LC3B (3868S), Ub (3936S), and active Caspase-3 (9664S) were purchased from Cell Signaling. Antibody against β-actin (sc-8432) was obtained from Santa Cruz. Antibody against RACK1 (610178) was from BD Biosciences. Antibody against electron-transferring-flavoprotein dehydrogenase (ETFDH) (A6585) was from ABclonal Biotech. Antibody against very long-chain acyl-CoA dehydrogenase (VLCAD) (14527-1-AP) was from Proteintech. Total protein extracts were prepared and dissolved in SDS sample buffer. Protein extracts were separated on 10% SDS-PAGE gels and transferred to polyvinylidene difluoride membranes (Millipore). The membranes were probed with antibodies and visualized with an electrochemiluminescence kit (Amersham).

### Generation of Bone Marrow Chimeras

Bone marrow cells were depleted of T cells and antigen-presenting cells by complement-mediated cell lysis. Bone marrow cells (2 × 10^6^) from 8-week-old *Gnb2l1*^F/F^ and *Gnb2l1*^F/F; CD4-Cre^ mice were cotransferred into sublethally irradiated recipients (9 Gy). The congenic markers CD45.1 and CD45.2 were used for distinguishing cells from the different donors and recipients.

### Purification of T Cell Subsets

Splenic CD4^+^ or CD8^+^ T cells were isolated by negative selection from single-cell suspensions of SPLs using a CD4^+^ T cell isolation kit II or a CD8^+^ T cell isolation kit II (Miltenyi Biotec). To get naïve CD4^+^ or CD8^+^ T cells, purified CD4^+^ or CD8^+^ splenic cells were stained with fluorescence-labeled antibodies against CD44 and CD62L. Then CD44^low^CD62L^high^ subsets were sorted using a FACSVantage (BD Biosciences). To purify thymic double negative (DN), double positive (DP), CD4 single positive (SP), and CD8 SP cells, single-cell suspensions of thymi were stained with fluorescence-conjugated antibodies against cell surface markers CD4 and CD8; different subsets were then sorted using a FACSVantage (BD Biosciences). In each case, purity of the cell separation was verified by flow cytometry analysis. Cell purity was verified to be at least 95%.

### Indirect Immunofluorescence Analysis of Autophagic LC3 Puncta

After treatment with 10 µM chloroquine (CQ) for 16 h, the cells were fixed and permeabilized using a fixation/permeabilization kit (eBioscience). The cells were then incubated with rabbit polyclonal antibody against LC3B (L7543; Sigma) for 1 h at room temperature, followed by incubation with FITC-conjugated goat anti-rabbit IgG for 30 min at room temperature. After incubation with 1 µg/ml 4′,6-diamidine-2-phenylindole (DAPI) for 5 min, the cells were observed under a laser scanning confocal microscopy (RADIANCE 2100; Bio-Rad).

### *In Vitro* Assays for T Cell Proliferation

Naïve CD4^+^ or CD8^+^ T cells were labeled by incubation at the density of 1.0 × 10^6^/ml in RPMI 1640 with 0.1 µM carboxyfluorescein succinimidyl ester (CFSE; Invitrogen) at 37°C for 20 min, washed, and resuspended in the complete culture medium. Cells were stimulated with Dynabeads mouse CD3/CD28 T cell expanders (Invitrogen) in 96-well plates at the density of 2.5 × 10^4^ cells/well. Proliferation was assessed by flow-cytometric analysis of CFSE dilutions after 72 h of culture.

### 5-Bromo-2′-Deoxyuridine (BrdU) Incorporation

Mice received 1 mg thymidine analog BrdU (Sigma) in 0.1 ml PBS via i.p. injection. BrdU incorporation in CD4^+^ or CD8^+^ splenic T cells was analyzed by flow cytometry 24 h later. Staining of BrdU incorporation followed the BrdU Flow Kit (Becton Dickinson) protocol. Briefly, cells were dehydrated in an alcohol solution, fixed and permeabilized in 1% paraformaldehyde/0.01% Tween 20, treated with 50 U/ml DNase I, and then stained with 10 µl of FITC-conjugated anti-BrdU (Becton Dickinson).

### Induction of Bone Marrow-Derived Macrophages (BMDM)

Bone marrow-derived macrophages were obtained by culturing the non-adherent bone marrow cells from 6- to 8-week-old mice in RPMI 1640 medium containing 15% (v/v) FBS, 2 mM l-glutamine, 100 U/ml penicillin, 100 mg/ml streptomycin, and 50 µM 2-ME with 100 ng/ml M-CSF for 7 days.

### Apoptosis

Purified CD4^+^ or CD8^+^ T cells were seeded into a 96-well plate at the density of 2.5 × 10^4^ cells/well. The cells were stimulated with Dynabeads mouse CD3/CD28 T cell expanders (Invitrogen) or left untreated. 0 or 24 h after culture without stimulation or 72 h after stimulation, cells were stained with Annexin V-FITC and PI resuspended in 300 µl binding buffer containing calcium ion. Apoptosis was assessed by flow-cytometric analysis.

### Measurement of Mitochondrial Content

To stain mitochondria, cells were incubated for 30 min at 37°C with 100 nM MitoTracker Green (Molecular Probes) in RPMI 1640 complete medium before staining of surface markers. Mitochondrial content was assessed by flow-cytometric analysis.

### Concanavalin A (Con A) Treatment

Male *Gnb2l1*^F/F; CD4-Cre^ mice at the age of 8–12 weeks and their littermates were intravenously injected with Con A (Sigma) dissolved in pyrogen-free saline at a dose of 25 µg/g body weight. Survival was monitored for 120 h after injection.

### Histological Assessment

Mice were euthanized, and livers were removed 48 h after Con A injection. The livers were fixed for another 48 h in 4% paraformaldehyde, dehydrated, infiltrated with paraffin, and sectioned at 5 µm. Slides were stained with hematoxylin/eosin.

### Assays for Plasma Transaminase Activity

Serum alanine transaminase (ALT) and aspartate transaminase (AST) levels were determined using commercially available kits (Abcam) according to the manufacturer’s protocols.

### Preparation of Liver Mononuclear Cells

Mice were euthanized, and livers were removed. Then, livers were pressed through a 200-gage stainless steel mesh. After the filtrate was washed once, the cells were resuspended in 40% Percoll (GE Healthcare) and overlaid onto 70% Percoll. After centrifugation at 1,260 *g* for 30 min, the interphase was collected and washed once.

### Statistics

Results are shown as mean ± SD. Differences were considered significant with a *P* value of <0.05 using rank sum test, Student’s *t*-test (paired or unpaired), or one-way analysis of variance (ANOVA).

## Results

### RACK1 Is Dispensable for Intrathymic Development of Conventional T Cells and Regulatory T (Treg) Cells

**Figure 1:**
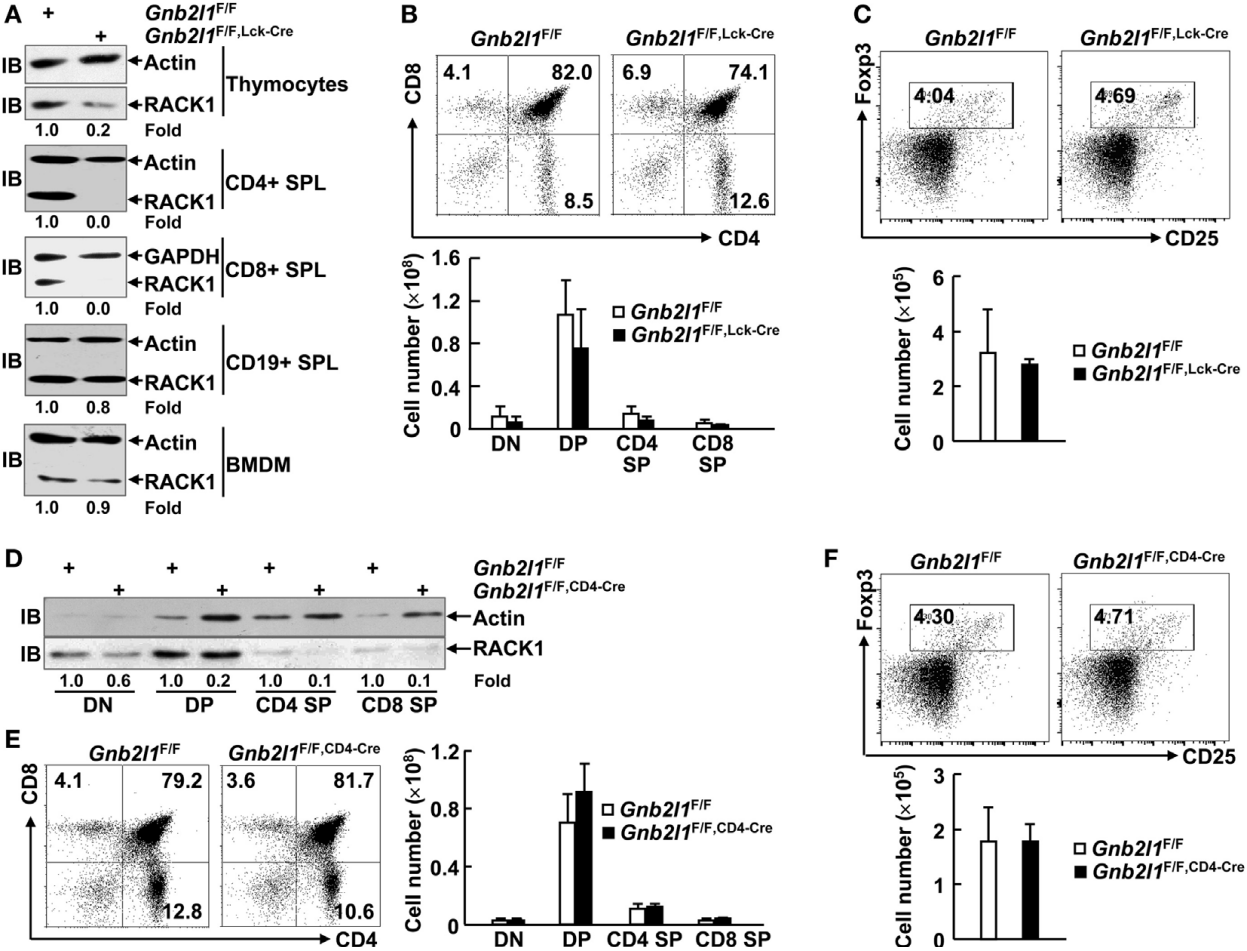
**Receptor for activated C kinase 1 (RACK1) is dispensable for intrathymic development of conventional T cells and regulatory T (Treg) cells**. **(A,D)** Immunoblotting (IB) analysis of the expression of RACK1 and β-actin in different immune cell subsets of 6- to 8-week-old *Gnb2l1*^F/F; lck-Cre^ mice **(A)** or *Gnb2l1*^F/F; CD4-Cre^ mice **(D)** as compared to littermate controls. SPL, spleen; BMDM, bone marrow-derived macrophages. Densitometric readings are shown for RACK1 and normalized with β-actin protein. **(B,E)** Flow-cytometric analysis of double negative (DN), double positive (DP), CD4 single positive (SP), and CD8 SP thymocytes from 6- to 8-week-old *Gnb2l1*^F/F; lck-Cre^ mice **(B)** or *Gnb2l1*^F/F; CD4-Cre^ mice **(E)** as compared to littermate controls. Representative plots (*Left*) and the mean absolute numbers ± SD (*Right*) of the indicated thymocyte subsets are shown (*n* = 6). Please note that no statistical difference was observed. **(C,F)** Flow-cytometric analysis of Treg cells in CD4 SP thymocytes from 6- to 8-week-old *Gnb2l1*^F/F; lck-Cre^ mice **(C)** or *Gnb2l1*^F/F; CD4-Cre^ mice **(F)** as compared to littermate controls. Representative plots (*Top*) and the mean absolute numbers ± SD (*Bottom*) are shown (*n* = 6). Please note that no statistical difference was observed.

Specific inactivation of RACK1 in T cells was first achieved by crossing mice homozygous for a *Gnb2l1* conditional allele (*Gnb2l1*^F/F^) with mice expressing a transgene encoding Cre recombinase driven by the lymphocyte-specific protein tyrosine kinase (*Lck*) proximal promoter (17). Genotyping confirmed the presence of *LoxP* sites and *lck-Cre* in *Gnb2l1*^F/F; lck-Cre^ mice (data not shown). These mutants were born at the expected Mendelian ratio and appeared phenotypically normal (data not shown). RACK1 deficiency in thymocytes and splenic T cells was confirmed by IB (Figure 1A). However, CD19^+^ B cells in the SPL and BMDM showed unaltered levels of RACK1 protein (Figure 1A). Comprehensive analyses of T cell production in the thymus revealed that the percentages and absolute numbers of DN, DP, and SP CD4^+^ and CD8^+^ thymocytes appeared to unaltered in *Gnb2l1*^F/F; lck-Cre^ mice compared with littermate controls (Figure 1B). Furthermore, intrathymic development of Treg cells expressing Foxp3 transcription factor was normal in *Gnb2l1*^F/F; lck-Cre^ mice (Figure 1C). To further confirm the abovementioned observations, we also generated a T cell-specific RACK1 knockout mouse model (*Gnb2l1*^F/F; CD4-Cre^ mice) with *CD4-Cre* transgenic mice (18, 19). IB analysis confirmed the deficiency of RACK1 in thymocytes, especially in CD4 SP and CD8 SP subsets (Figure 1D). Consistent with the data obtained in *Gnb2l1*^F/F; lck-Cre^ mice, the dispensable role of RACK1 in intrathymic development of conventional T cells and Treg cells was also observed in *Gnb2l1*^F/F; CD4-Cre^ mice (Figures 1E,F).

### Specific Deletion of RACK1 in T Cells Leads to Peripheral T Cell Lymphopenia

**Figure 2:**
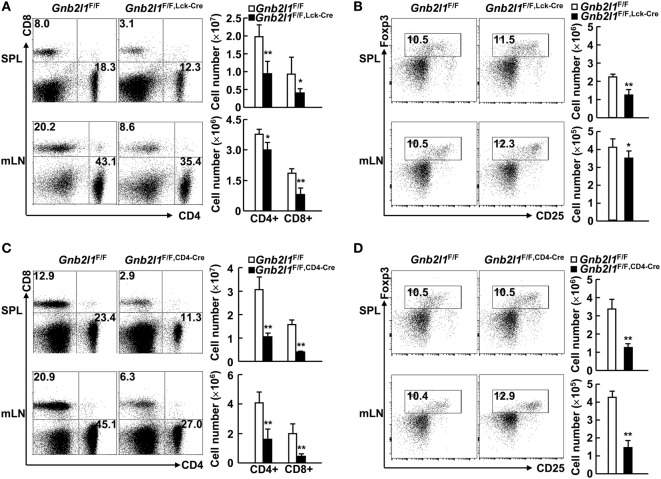
**Specific deletion of receptor for activated C kinase 1 (RACK1) in T cells leads to peripheral T cell lymphopenia**. **(A,C)** Flow-cytometric analysis of CD4^+^ and CD8^+^ T cells in the spleen (SPL) and mesenteric lymph nodes (mLNs) of 6- to 8-week-old *Gnb2l1*^F/F; lck-Cre^ mice **(A)** or *Gnb2l1*^F/F; CD4-Cre^ mice **(C)** as compared to littermate controls. Representative plots (*Left*) and the mean absolute numbers ± SD (*Right*) of the indicated T cell subsets are shown (*n* = 6). *P < 0.05 and **P < 0.01. **(B,D)** Flow-cytometric analysis of regulatory T (Treg) cells in peripheral CD4^+^ T cells of 6- to 8-week-old *Gnb2l1*^F/F; lck-Cre^ mice **(B)** or *Gnb2l1*^F/F; CD4-Cre^ mice **(D)** as compared to littermate controls. Representative plots (*Left*) and the mean absolute numbers ± SD (*Right*) are shown (*n* = 6). Please note that no statistical difference was observed.

We next examined the peripheral T lymphocyte compartment in *Gnb2l1*^F/F; lck-Cre^ mice and *Gnb2l1*^F/F; CD4-Cre^ mice. The percentages and absolute numbers of CD4^+^ and CD8^+^ T cells in the SPL and mLNs of *Gnb2l1*^F/F; lck-Cre^ mice and *Gnb2l1*^F/F; CD4-Cre^ mice were significantly reduced (Figures 2A,C). The general reduction in peripheral T cells was more profound in *Gnb2l1*^F/F; CD4-Cre^ mice than that in *Gnb2l1*^F/F; lck-Cre^ mice (Figures 2A,C). However, the percentages of Treg cells in peripheral CD4^+^ T cells remained unaffected in the absence of RACK1 (Figures 2B,D) even though their absolute numbers decreased proportionally due to the reduced numbers of peripheral CD4^+^ T cells (Figures 2B,D).

### CD8^+^ T Cells, But Not CD4^+^ T Cells, Tend to Show Enhanced Activation/Memory in the Absence of RACK1

**Figure 3:**
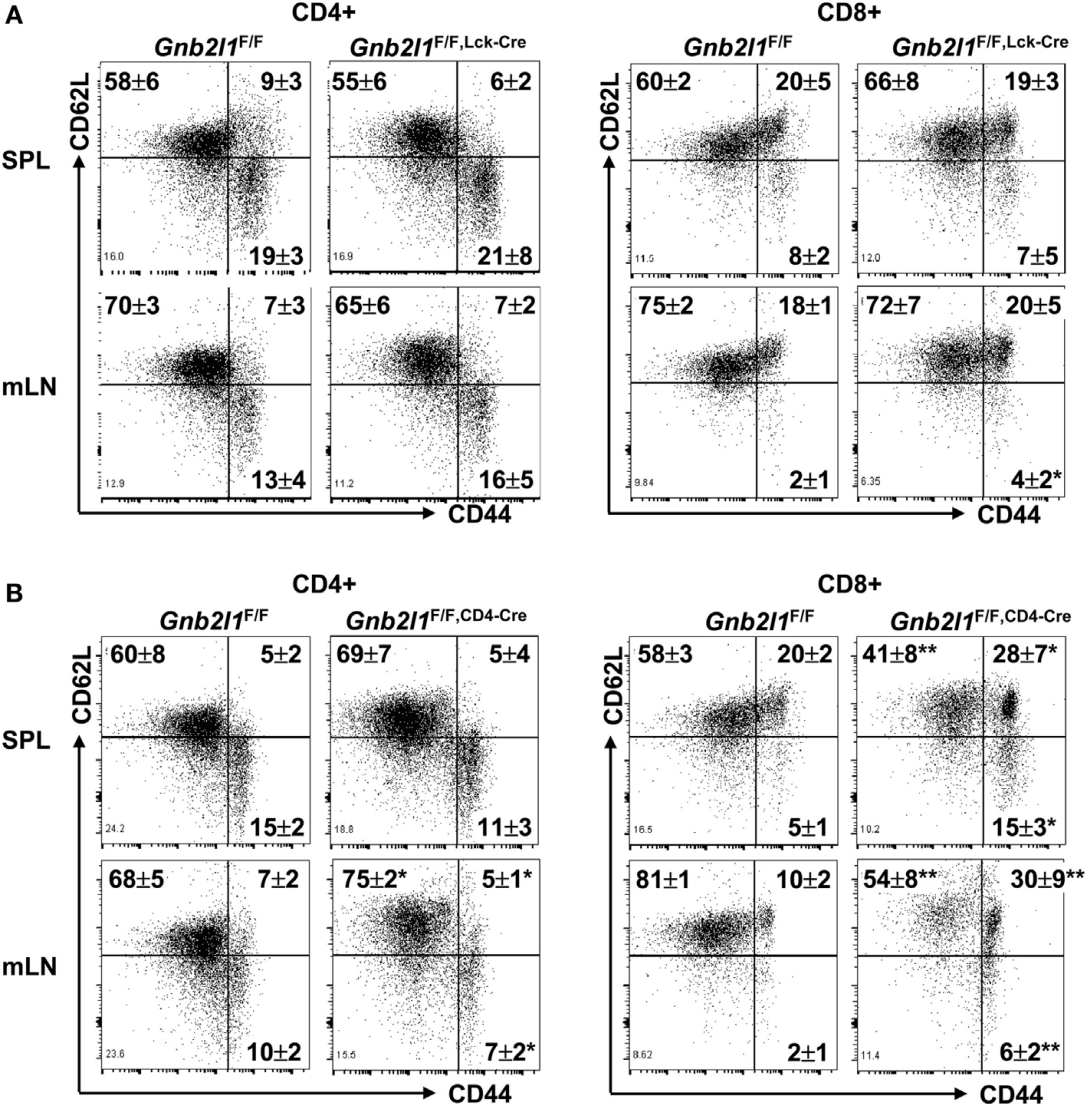
**CD8^+^ T cells, but not CD4^+^ T cells, tend to show enhanced activation/memory in the absence of receptor for activated C kinase 1 (RACK1)**. Flow-cytometric analysis of the expression of CD44 and CD62L in peripheral CD4^+^ and CD8^+^ T cells of 6- to 8-week-old *Gnb2l1*^F/F; lck-Cre^ mice **(A)** or *Gnb2l1*^F/F; CD4-Cre^ mice **(B)** as compared to littermate controls. Representative plots and the mean percentages ± SD are shown (*n* = 6). *P < 0.05 and **P < 0.01.

In lymphopenic mice, residual peripheral T cells usually express a marker profile characteristic of either activated effector T cells or central memory T cells (20, 21). The lymphopenia upon specific deletion of Atg3, Atg5, Atg7, Atg16l1, or Vps34 in T cells has been demonstrated to be associated with increased percentages of activation/memory-like T cells within both the CD4^+^ and CD8^+^ compartments (2–6). On the other hand, splenic CD4^+^ T cells did not show changed expression profile of CD44 and CD62L upon specific deletion of Beclin 1 in T cells, even though more splenic CD8^+^ T cells were CD44^high^CD62L^low^ in the absence of Beclin 1 (7). In this scenario, we analyzed the expression profile of CD44 and CD62L in the presence and absence of RACK1 in T cells. CD4^+^ T cells in the SPL and mLNs of 6- to 8-week-old *Gnb2l1*^F/F; lck-Cre^ mice (Figure 3A) and *Gnb2l1*^F/F; CD4-Cre^ mice (Figure 3B) showed no increased percentage of CD44^high^CD62L^low^ or CD44^high^CD62L^high^ population as compared to their counterparts in littermates. Splenic CD8^+^ T cells of *Gnb2l1*^F/F; lck-Cre^ mice also demonstrated no enhanced activation/memory, whereas CD8^+^ T cells in the mLNs of *Gnb2l1*^F/F; lck-Cre^ mice exhibited a slight increase in the frequency of CD44^high^CD62L^low^ population (Figure 3A). On the other hand, CD8^+^ T cells in the SPL and mLNss of *Gnb2l1*^F/F; CD4-Cre^ mice all showed higher percentages of CD44^high^ CD62L^low^ and CD44^high^CD62L^high^ populations (Figure 3B). However, CD8^+^ T cells in the SPL and mLNs of *Gnb2l1*^F/F; CD4-Cre^ mice showed only slightly enhanced CD69 and CD25 expression under steady-state conditions (Figure S1 in Supplementary Material). Therefore, RACK1-deficient CD8^+^ T cells *in vivo* may be responding to homeostatic rather than antigen-induced expansion signals, which result in aberrant expression profile of CD44 and CD62L.

### Peripheral T Cell Lymphopenia upon Specific Deletion of RACK1 in T Cells Results from Certain Cell-Intrinsic Defect(s)

**Figure 4:**
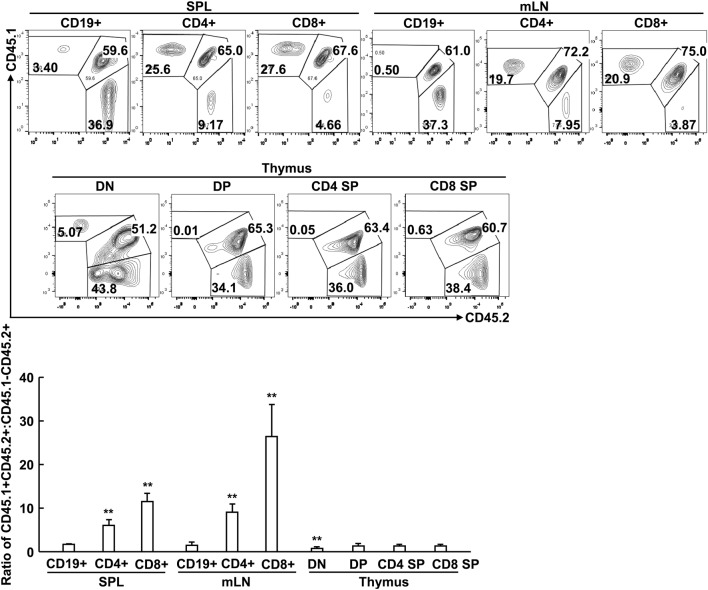
**Peripheral T cell lymphopenia in the absence of receptor for activated C kinase 1 (RACK1) results from certain cell-intrinsic defect(s)**. Mixed bone marrow chimeras were used after 8 weeks of bone marrow reconstitution. Flow-cytometric analysis of the relative contributions of CD45.1^+^CD45.2 and CD45.1^−^CD45.2^+^ bone marrow cells to the regeneration of different immune cell subsets in spleen (SPL), mesenteric lymph node (mLN), and thymus of chimeric mice. Representative plots of gated immune cell subsets (*Top*). The mean ratios ± SD of CD45.1^+^CD45.2^+^ vs. CD45.1^−^CD45.2^+^ (*n* = 3) (*Bottom*). Data shown in this figure are representative of at least three independent experiments. *P < 0.05 and **P < 0.01.

To distinguish T cell-intrinsic or -extrinsic mechanism(s) under-lying peripheral T cell lymphopenia upon specific deletion of RACK1 in T cells, we created mice with mixed bone marrow through transferring bone marrow cells from congenically marked *Gnb2l1*^F/F^ (CD45.1CD45.2) and *Gnb2l1*^F/F; CD4-Cre^ (CD45.2CD45.2) mice into sublethally irradiated CD45.1CD45.1 mice. We chose *Gnb2l1*^F/F; CD4-Cre^ mice because peripheral T cell lymphopenia in these mice is more profound than that in *Gnb2l1*^F/F; lck-Cre^ mice. After 8 weeks of bone marrow reconstitution, the CD19^+^ B-cell compartment showed a 1.62:1 ratio of CD45.1^+^CD45.2^+^ vs. CD45.1^−^CD45.2^+^ (Figure 4). Similar ratios were observed in DP, CD4 SP, and CD8 SP thymocyte subsets (Figure 4). However, peripheral T cells were overwhelmingly derived from RACK1-sufficient CD45.1^+^CD45.2^+^ cells (Figure 4). Interestingly, RACK1 was more important for the reconstitution of peripheral CD8^+^ T cells than peripheral CD4^+^ T cells (Figure 4). All of these findings are consistent with the phenotype of our *Gnb2l1*^F/F; lck-Cre^ mice and *Gnb2l1*^F/F; CD4-Cre^ mice in the steady state and suggest that RACK1 plays a crucial cell-autonomous role in maintaining the peripheral T cell number.

### RACK1 Absence Leads to Impaired Basal Autophagy and the Accumulation of Mitochondria

**Figure 5:**
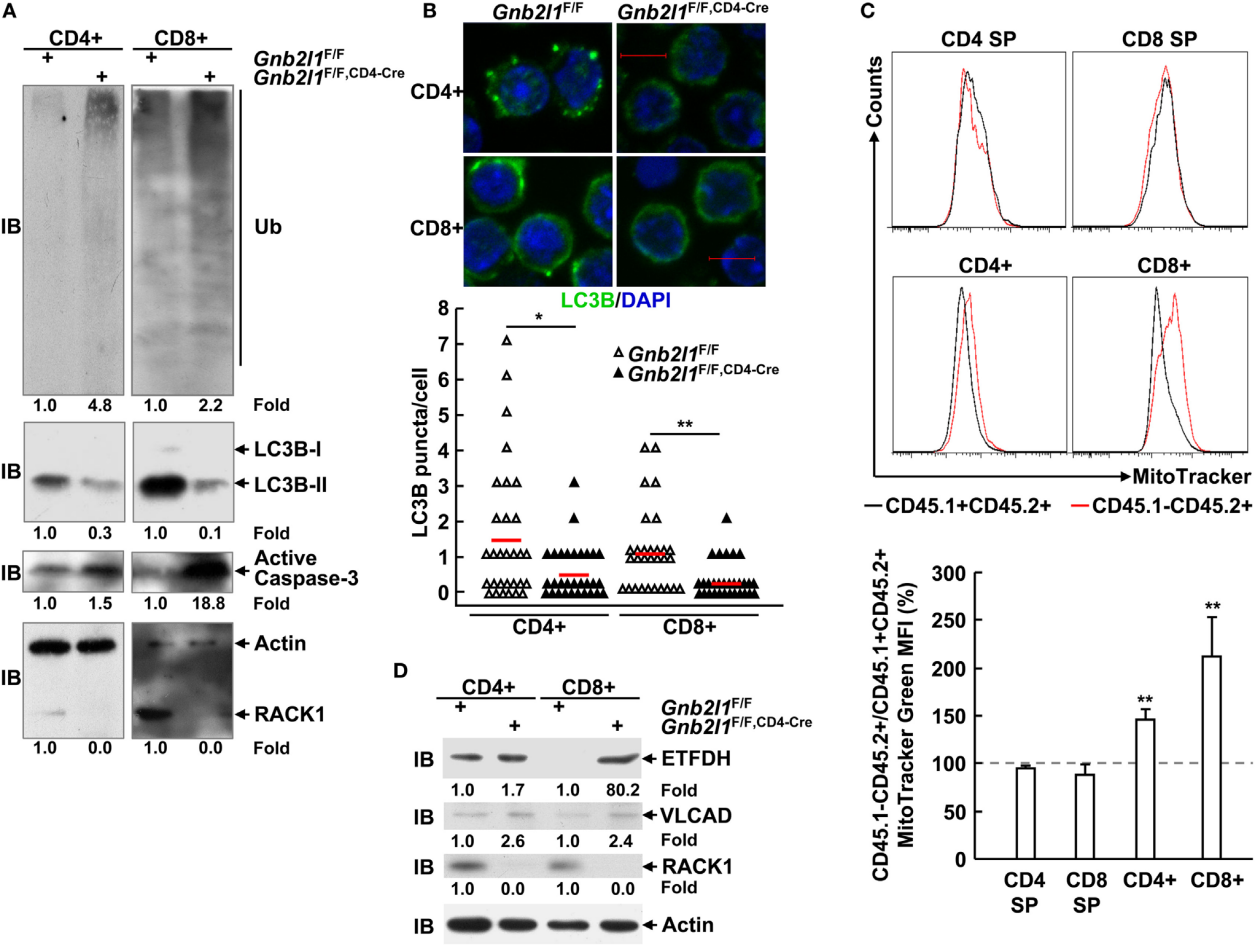
**Receptor for activated C kinase 1 (RACK1) absence leads to impaired basal autophagy and the accumulation of mitochondria**. **(A,D)** Splenic T cells purified from 6- to 8-week-old *Gnb2l1*^F/F; CD4-Cre^ mice and littermate controls were subjected to immunoblotting (IB) analysis. Densitometric readings are shown for polyubiquitinated proteins, LC3B-II, active Caspase-3, ETFDH, VLCAD, and RACK1 and normalized with β-actin protein. Ub, ubiquitin; ETFDH, electron-transferring-flavoprotein dehydrogenase; VLCAD, very long-chain acyl-CoA dehydrogenase. **(B)** Peripheral T cells isolated from 6- to 8-week-old *Gnb2l1*^F/F; CD4-Cre^ mice and littermate controls were treated with 10 µM chloroquine (CQ) for 16 h, followed by indirect immunofluorescence analysis of endogenous LC3B. Representative pictures of the LC3B staining (scale bar, 5 µm) (*Top*). A total of 30 cells were counted (*Bottom*). *P < 0.05 and **P < 0.01. **(C)** Mixed bone marrow chimeras were used after 8 weeks of bone marrow reconstitution. Mitochondrial content in RACK1-sufficient (CD45.1^+^CD45.2^+^) and -deficient (CD45.1^−^CD45.2^+^) T cell subsets was assessed by flow-cytometric analysis after single-cell suspensions of thymi, and SPLs were incubated with MitoTracker Green. Representative plots of gated CD45.1^+^CD45.2^+^ and CD45.1^−^CD45.2^+^ subsets (*Top*). The mean percentages ± SD of MitoTracker Green mean fluorescent intensity (MFI) in gated CD45.1^−^CD45.2^+^ vs. CD45.1^+^CD45.2^+^ subsets (*n* = 3) (*Bottom*). Data shown in this figure are representative of at least three independent experiments. *P < 0.05 and **P < 0.01.

We have shown that RACK1 promotes autophagy in several types of mammalian cells *in vivo* and *ex vivo* (15). The impaired peripheral T lymphocyte compartment in RACK1-deficient mice was similar to that in mice lacking autophagy genes (1–9). It is possible that peripheral T cell lymphopenia in *Gnb2l1*^F/F; lck-Cre^ mice and *Gnb2l1*^F/F; CD4-Cre^ mice was due to impaired autophagy. In line with this notion, IB analysis revealed that splenic T cells from *Gnb2l1*^F/F; CD4-Cre^ mice showed diminished basal membrane-bound LC3B-II generation from cytosolic LC3B-I (Figure 5A), an indicator of autophagic activity (22, 23). Consistently, indirect immunofluorescence analysis demonstrated that peripheral T cells from *Gnb2l1*^F/F; CD4-Cre^ mice exhibited reduced numbers of LC3B-positive dots (autophagic LC3 puncta) after treatment with lysosomal protease inhibitor CQ (Figure 5B). Impaired autophagy onset was associated with the accumulation of polyubiquitinated proteins and enhanced production of active Caspase-3 fragment (Figure 5A).

Previous reports have demonstrated that autophagy limits the number of mitochondria in peripheral T cells (2–4, 6). As expected, RACK1-deficient CD8^+^ splenic T cells from the bone marrow chimeric mice had increased mitochondrial mass, as determined by staining with a cell-permeable mitochondrial dye (MitoTracker) (Figure 5C). RACK1-deficient CD4^+^ T cells also showed an increase in mitochondrial mass, albeit to a lesser extent (Figure 5C). Interestingly, RACK1-sufficient and -deficient CD4 SP and CD8 SP thymocytes exhibited comparable MitoTracker Green staining (Figure 5C), suggesting a specific role for RACK1 in peripheral T cell homeostasis but not T cell development. The same phenomena were also observed in RACK1-deficient T cells isolated from *Gnb2l1*^F/F; CD4-Cre^ mice as compared to their counterparts isolated from littermate controls (Figure S2 in Supplementary Material). Confocal microscopy also revealed that RACK1-deficient T cells had more MitoTracker-positive structures (Figure S3A in Supplementary Material). Furthermore, the MitoTracker-positive structures in RACK1-deficient cells frequently formed tubular clusters (Figure S3A in Supplementary Material). Ultrastructural analysis confirmed increased numbers of mitochondria and revealed certain elongated mitochondria in RACK1-deficient CD8^+^ T cells. In addition, electron microscopy showed that some membrane structures that are not characteristic of autophagosomes accumulated in RACK1-deficient CD8^+^ T cells (Figure S3B in Supplementary Material), similar to the observations in Atg3-deficient T cells (4). Consistently, IB analysis revealed that mitochondrial proteins ETFDH (electron transfer flavoprotein dehydrogenase) and VLCAD (very-long-chain acyl-CoA dehydrogenase) (24, 25) were accumulated in splenic T cells from *Gnb2l1*^F/F; CD4-Cre^ mice (Figure 5D).

### Specific Deletion of RACK1 in T Cells Leads to Cell-Intrinsic Defects in Cell Survival and Proliferation

**Figure 6:**
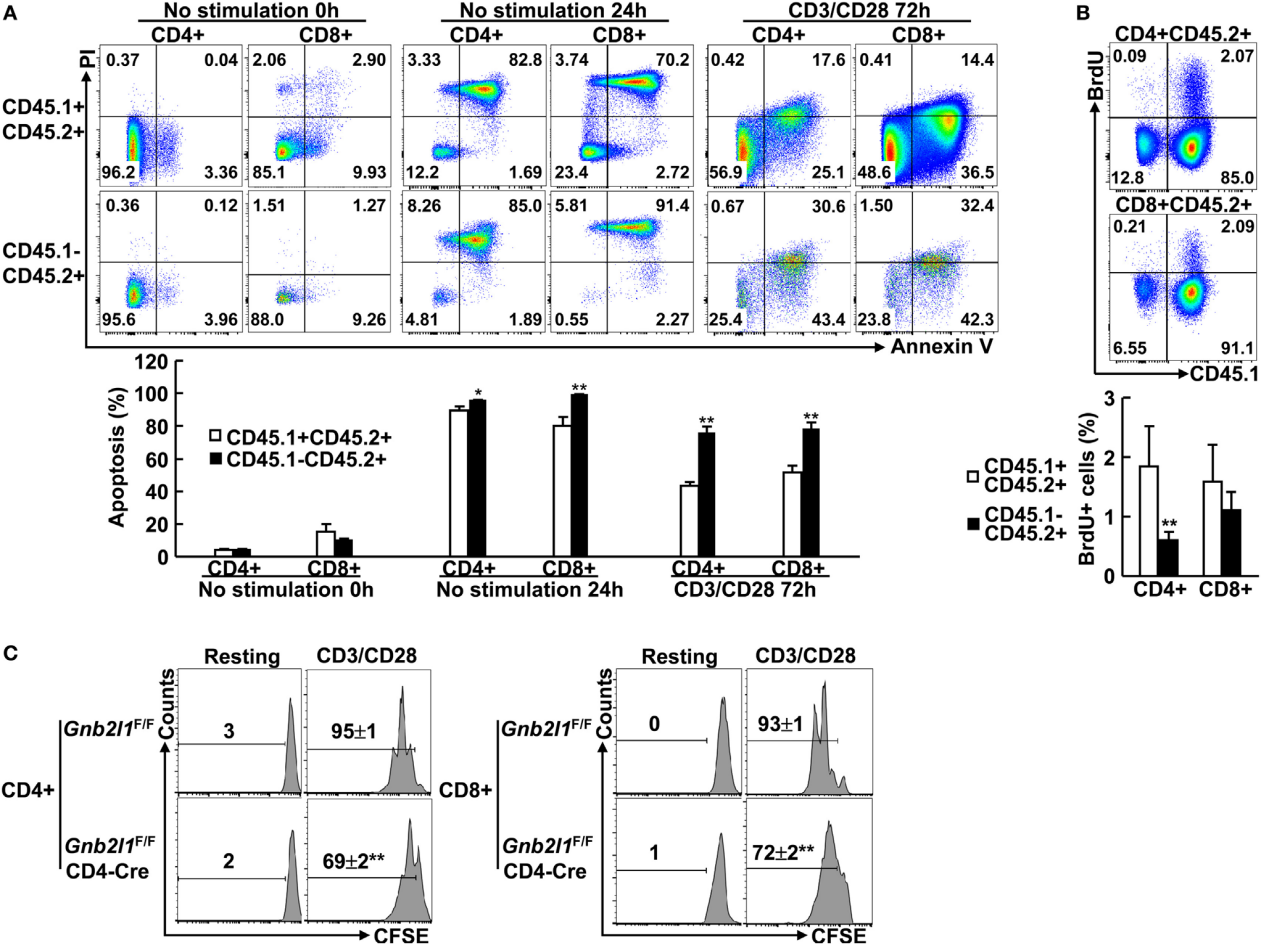
**Specific deletion of receptor for activated C kinase 1 (RACK1) in T cells leads to cell-intrinsic defects in cell survival and proliferation**. **(A,B)** Mixed bone marrow chimeras were used after 8 weeks of bone marrow reconstitution. Purified CD4^+^ or CD8^+^ T cells were stimulated with Dynabeads mouse CD3/CD28 T cell expanders or left untreated. In all, 0 or 24 h after culture without stimulation or 72 h after stimulation, cells were stained with Annexin V-FITC and PI. Apoptosis was assessed by flow-cytometric analysis **(A)**. The chimeric mice received 5-bromo-2′-deoxyuridine (BrdU) via i.p. injection. BrdU incorporation in CD4^+^ or CD8^+^ splenic T cells was analyzed by flow cytometry 24 h later **(B)**. Representative plots of gated subsets (*Top*). The mean percentages ± SD of apoptosis **(A)** or BrdU^+^ cells **(B)** in the indicated subsets (*n* = 3) (*Bottom*). Data shown in this figure are representative of at least three independent experiments. *P < 0.05 and **P < 0.01. **(C)** Naïve CD4^+^ and CD8^+^ T cells purified from 6- to 8-week-old *Gnb2l1*^F/F; CD4-Cre^ mice and littermate controls were labeled with carboxyfluorescein succinimidyl ester (CFSE). After stimulation with Dynabeads mouse CD3/CD28 T cell expanders for 72 h, cells were stained with Annexin V and subjected to flow cytometry analysis. Annexin V-negative cells were gated. Representative plots and the mean percentages ± SD of cells with diluted CFSE are shown (*n* = 4). *P < 0.05 and **P < 0.01.

Autophagy-deficient T cells readily undergo apoptosis because healthy mitochondria are crucial for T cell survival (2–4, 6). Thus, it is of importance to examine whether RACK1-deficient T cells had increased apoptosis. To exclude cell-extrinsic effects (e.g., activation-induced cell death due to lymphopenia), we used mixed bone marrow chimeras. In these mixed chimeras, RACK1-sufficient and -deficient splenic T cells exhibited similar basal apoptosis *ex vivo* (Figure 6A). However, RACK1-deficient splenic T cells were more vulnerable than their RACK1-sufficient counterparts after *in vitro* culture in exactly the same system (Figure 6A). Moreover, more RACK1-deficient splenic T cells underwent apoptosis than their RACK1-sufficient counterparts did upon TCR ligation (Figure 6A). These data suggest a cell-intrinsic requirement for RACK1 in maintaining peripheral T cell survival.

Besides increased sensitivity to cell death, the reduced numbers of peripheral T cells in the absence of RACK1 might also result from decreased proliferation potential since previous results have indicated that Atg3-, Atg5-, Atg7- or Vps34-deficient T cells cannot proliferate efficiently (2–4, 8, 9). Indeed, RACK1-deficient CD4^+^ T cells in the chimeras, but not CD8^+^ T cells, showed reduced BrdU incorporation as compared to RACK1-sufficient counterpart in the steady state (Figure 6B). Next, we tested how RACK1-deficient T cells respond to TCR-mediated stimulation. For this purpose, naïve CD4^+^ and CD8^+^ T cells were purified from *Gnb2l1*^F/F; CD4-Cre^ mice and their control littermates and labeled with the fluorescent-dye CFSE. After 3 days of anti-CD3 and anti-CD28 stimulation, the cells were subjected to flow cytometry analysis of CFSE dilution. To exclude the possibility that the possible proliferation defect is caused by cell death, all cells in the CFSE dilution assay were gated on Annexin V-negative cells. We found that both CD4^+^ and CD8^+^ T cells proliferated less efficiently in the absence of RACK1 (Figure 6C). Despite that, *in vitro* differentiation of naïve CD4^+^ T cells isolated from *Gnb2l1*^F/F; CD4-Cre^ mice was not impaired under Th1 polarization conditions as compared with their counterparts isolated from control littermates (Figure S4 in Supplementary Material).

### RACK1 Is Required for Invariant Natural Killer T (iNKT) Cell Development

**Figure 7:**
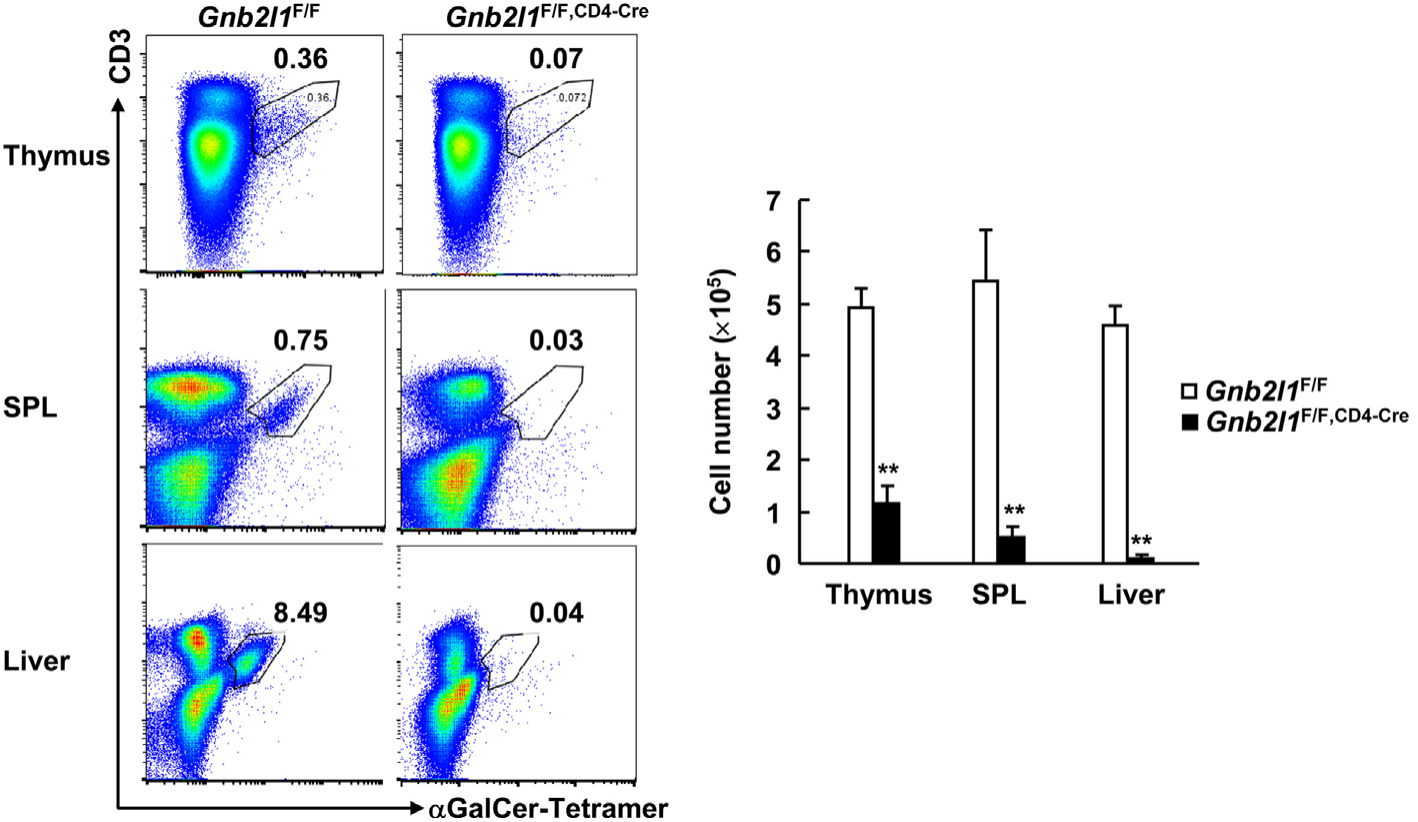
**Receptor for activated C kinase 1 (RACK1) is required for invariant natural killer T (iNKT) cell development**. Mononuclear cells from thymi, spleens (SPLs), and livers of 6- to 8-week-old *Gnb2l1*^F/F; CD4-Cre^ mice and littermate controls were stained with fluorescence-labeled antibodies against CD3 and CD19 and αGalCer-loaded CD1d-tetramer, followed by flow-cytometric analysis. CD19-negative live cells were gated. Representative plots (*Left*) and the mean absolute numbers ± SD (*Right*) are shown (*n* = 4). *P < 0.05 and **P < 0.01.

Invariant natural killer T cells are a small population of T cells whose development in the thymus requires expression of the Vα14-Jα18 invariant TCR and its recognition of endogenous glycolipid ligands presented by CD1d expressed on DP thymocytes (8, 26, 27). After their development, these cells populate peripheral lymphoid organs, mainly liver and SPL (8, 26, 27). An essential role for autophagy during iNKT cell development has been disclosed (8, 26, 27). In this scenario, it is of interest to explore how T cell-specific RACK1 deficiency might affect iNKT cell development. As expected, we found that *Gnb2l1*^F/F; CD4-Cre^ mice exhibited an obvious defect in intrathymic iNKT cell development; only about 20% CD3^+^CD1d-tetramer^+^ cells were observed as compared with control littermates (Figure 7). *Gnb2l1*^F/F; CD4-Cre^ mice showed more profound defect in the iNKT cell population in the SPL and liver as compared to their control littermates (Figure 7). Thus, thymic iNKT cell development is blocked and mature iNKT cells are absent in peripheral lymphoid organs in the absence of RACK1.

### T Cell-Specific Loss of RACK1 Dampens Con A-Induced Acute Liver Injury

**Figure 8:**
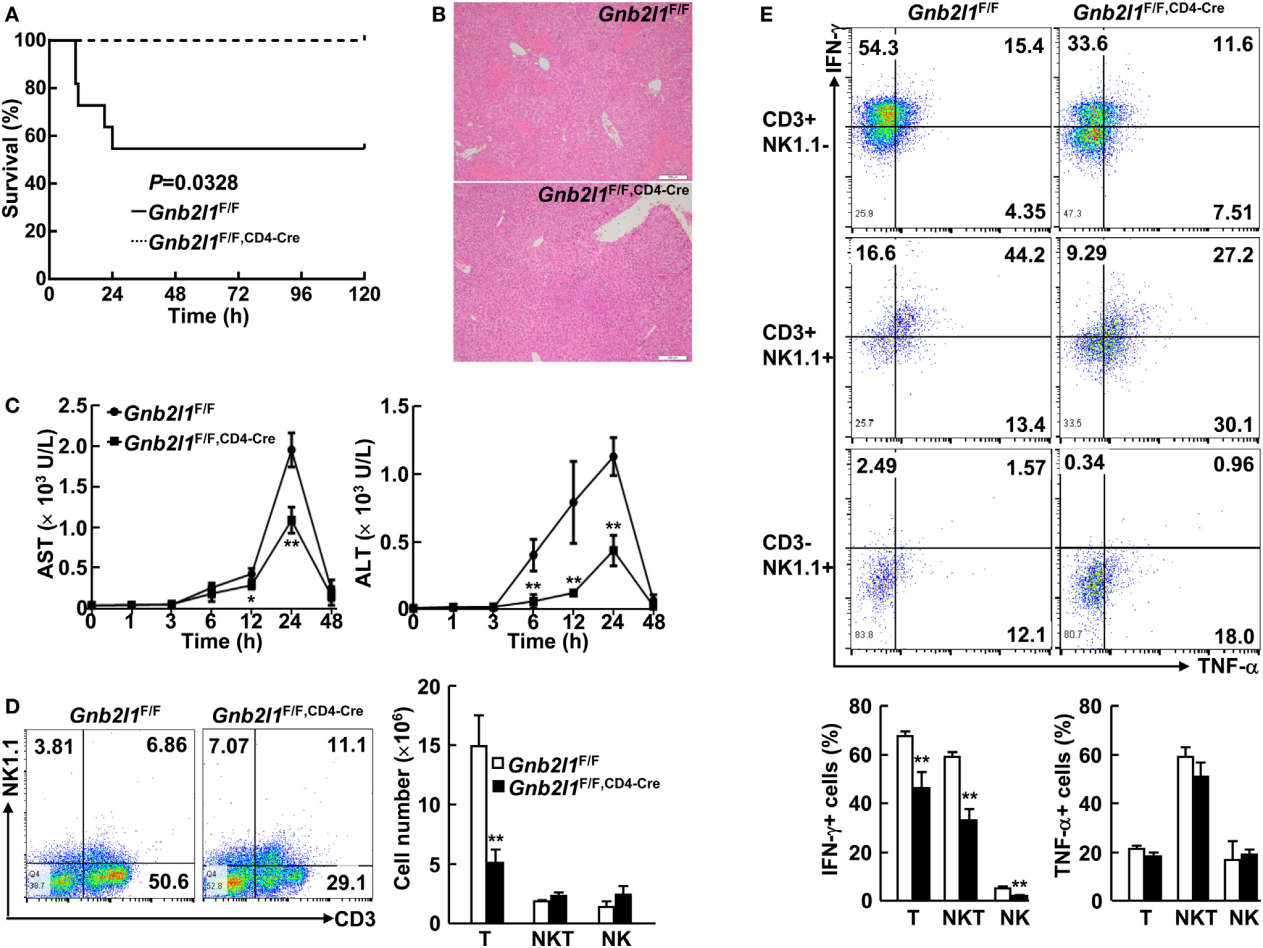
**T cell-specific loss of receptor for activated C kinase 1 (RACK1) dampens concanavalin A (Con A)-induced acute liver injury**. **(A)** *Gnb2l1*^F/F; CD4-Cre^ mice (*n* = 8) and their littermates (*n* = 11) at the age of 8–12 weeks were injected with Con A (25 µg/g body weight). The survival was monitored in 120 h. **(B)** Representative images of hematoxylin and eosin-stained liver sections from *Gnb2l1*^F/F; CD4-Cre^ mice and their littermates 48 h after Con A injection. Scale bar: 200 µm. **(C)** Serum aspartate transaminase (AST) and alanine transaminase (ALT) levels were measured at various time periods post Con A injection. Mean values ± SD are shown (*n* = 4). *P < 0.05 and **P < 0.01. **(D)** Flow-cytometric analysis of CD3^+^NK1.1^−^ T cells, CD3^+^NK1.1^+^ NKT cells, and CD3^−^NK1.1^+^ NK cells in the liver of *Gnb2l1*^F/F; CD4-Cre^ mice 2 h post Con A treatment as compared to littermate controls. Representative plots (*Left*) and the mean absolute numbers ± SD (*Right*) of the indicated lymphocyte subsets are shown (*n* = 6). *P < 0.05 and **P < 0.01. **(E)** Flow-cytometric analysis of IFN-γ or TNF-α production in the indicated lymphocyte subsets of *Gnb2l1*^F/F; CD4-Cre^ mice 2 h post Con A treatment as compared to littermate controls. Representative plots (*Top*) and the mean percentages ± SD (*Bottom*) are shown (*n* = 6). *P < 0.05 and **P < 0.01.

Our data indicate that specific deletion of RACK1 in T cells leads to peripheral T cell lymphopenia and impaired iNKT cell development. In this scenario, we tested how T cell-specific RACK1 deficiency might affect Con A-induced acute liver injury because T and NKT cells play essential roles in this model (28–30). A high dose of Con A (25 µg/g body weight) was injected into *Gnb2l1*^F/F; CD4-Cre^ mice and their littermates, and their survival was monitored. As expected, *Gnb2l1*^F/F; CD4-Cre^ mice all survived under the conditions that almost half of their littermates died (Figure 8A). Histological analysis revealed that *Gnb2l1*^F/F; CD4-Cre^ mice had very limited liver damage, while their littermates had extensive liver necrosis (Figure 8B). Measurement of serum AST and ALT levels also indicated that *Gnb2l1*^F/F; CD4-Cre^ mice had significantly reduced liver injury (Figure 8C).

Various cytokines, including IFN-γ and TNF-α, are involved in the pathogenesis of Con A-induced hepatitis (28–30), so we wondered whether RACK1 deficiency affects cytokine production. As shown in Figure 8D, *Gnb2l1*^F/F; CD4-Cre^ mice had reduced percentages and absolute numbers of CD3^+^ NK1.1^−^ T cells in the liver after Con A treatment as compared to their littermates. Furthermore, the percentage of IFN-γ^+^ T cells in *Gnb2l1*^F/F; CD4-Cre^ livers after Con A injection was significantly downregulated, while Con A-induced production of TNF-α in these cells was only slightly affected in *Gnb2l1*^F/F; CD4-Cre^ livers (Figure 8E). Surprisingly, *Gnb2l1*^F/F; CD4-Cre^ mice and control littermates showed comparable absolute numbers of CD3^+^NK1.1^+^ NKT cells as well as CD3^−^NK1.1^+^ NK cells in the liver after Con A treatment (Figure 8D), even though both subsets exhibited reduced IFN-γ expression (Figure 8E). In this scenario, we examined the relationship between CD3^+^NK1.1^+^ subset and CD3^+^CD1d-tetramer^+^ subset in the liver of *Gnb2l1*^F/F; CD4-Cre^ mice and control littermates. As previously reported (29, 30), most NKT cells in the liver of *Gnb2l1*^F/F^ mice were CD1d-tetramer^+^NK1.1^+^ iNKT cells under steady-state conditions when CD3^+^ cells were gated (Figure S5 in Supplementary Material). After Con A treatment, the percentage of CD1d-tetramer^+^NK1.1^+^ iNKT cells promptly dropped, whereas that of CD1d-tetramer^−^NK1.1^+^ NKT cells remained largely unchanged (Figure S5 in Supplementary Material). By contrast, there were very few CD1d-tetramer^+^NK1.1^+^ iNKT cells but significantly increased percentage of CD1d-tetramer^−^NK1.1^+^ NKT cells in the liver of *Gnb2l1*^F/F; CD4-Cre^ mice in the steady state (Figure S5 in Supplementary Material). After Con A treatment, the percentage of CD1d-tetramer^−^NK1.1^+^ NKT cells further increased (Figure S5 in Supplementary Material), which might account for the equal absolute numbers of total NKT cells in the liver of *Gnb2l1*^F/F; CD4-Cre^ mice and control littermates.

## Discussion

This work suggests a critical role of RACK1 in maintaining peripheral T cell numbers and iNKT cell development. In mechanism, we have provided evidence that RACK1 is required for autophagy in T cells (15). Autophagy is not required for intrathymic conventional T cell development after DN stage but is essential for iNKT cell development (1–9, 26, 27). In peripheral organs, autophagy is indispensable for the survival and efficient proliferation of peripheral T cells (1–9). Consistently, we have found RACK1-deficient splenic T cells show enhanced production of active Caspase-3 fragment and undergo enhanced apoptosis upon TCR ligation or *in vitro* culture. Moreover, RACK1-deficient splenic T cells cannot proliferate efficiently. In addition, RACK1 is essential for iNKT cell development. The requirement of RACK1 for T cell homeostasis is not likely related to any non-autophagic function. Overall, our findings strengthen the concept that bona fide autophagy is crucial for T cell homeostasis.

Mitochondrial content is developmentally regulated in T cells. Exit from the thymus marks a transition from high mitochondrial content in thymocytes to lower mitochondrial content in mature T cells (2–4). Autophagy has been proposed to maintain the survival of resting peripheral T cells mainly by reducing their mitochondrial content, and autophagy-deficient peripheral T cells fail to reduce their mitochondrial content *in vivo* (2–4, 6). Indeed, our data have also demonstrated that SP thymocytes, especially CD8 SP thymocytes, exhibited higher mitochondrial contents than their peripheral counterparts. Interestingly, RACK1 seems to prevent the accumulation of mitochondria in peripheral T cells but not in CD4 SP and CD8 SP thymocytes. The accumulation of mitochondria in CD8^+^ peripheral T cells is more profound than that in CD4^+^ peripheral T cells in the absence of RACK1, which might explain why the impact of RACK1 deletion on CD8^+^ peripheral T cells is more profound than its effect on CD4^+^ peripheral T cells. Overall, these findings suggest that RACK1-dependent autophagy is required for peripheral T cell survival by regulating mitochondrial homeostasis.

Specific deletion of Atg3, Atg5, Atg7, Atg16l1, or Vps34 in T cells has been demonstrated to lead to higher percentages of activation/memory-like peripheral T cells (2–6). However, this is not the case for specific deletion of RACK1 in T cells. Even though 6- to 8-week old *Gnb2l1*^F/F; CD4-Cre^ mice showed higher percentages of CD44^high^CD62L^low^ and CD44^high^CD62L^high^ CD8^+^ T cells, such changes are very weak in *Gnb2l1*^F/F; lck-Cre^ mice at the same age. By contrast, CD4^+^ T cells in *Gnb2l1*^F/F; lck-Cre^ mice and *Gnb2l1*^F/F; CD4-Cre^ mice showed no signs of enhanced activation/memory. The phenotype is similar to that upon specific deletion of Beclin 1 in T cells (7). Enhanced T cell activation/memory in the absence of Atg3, Atg5, Atg7, Atg16l1, or Vps34 can be partially attributed to peripheral T cell lymphopenia. Moreover, it is possible that the malfunction of Treg cells also plays a role. Atg5, Atg7, Atg16l1, or Vps34-mediated autophagy has been demonstrated to support the lineage stability and survival fitness of Treg cells, an immune subset controlling tolerance and inflammation (5, 8, 31). Selective deletion of Atg16l or Vps34 in T cells results in the development of inflammatory disorders (5, 8). Our *Gnb2l1*^F/F; lck-Cre^ mice and *Gnb2l1*^F/F; CD4-Cre^ mice, however, look normal even at the age of 18 months (data not shown). We have shown that RACK1 promotes autophagy through enhancing Atg14L–Beclin 1–Vps34–Vps15 complex formation (15). It is possible that some non-classical autophagy independent of the autophagy-initiation complex maintains the lineage stability and survival fitness of Treg cells. Indeed, Beclin 1-independent autophagy has been reported by a few groups (32–36). It is also possible that certain binding partner(s) of RACK1 other than the autophagy-initiation complex compensates the defects originated from impaired autophagy in Treg cells. In line with this notion, it has been suggested that RACK1 is involved in intracellular Ca^2+^ influx in Treg cells (37). Besides, RACK1 may anchor Lck, PKC, and GRID (Grb2-related protein with insert domain) to function in T cell signaling pathways (38, 39). Future studies are required to address how RACK1 affects Treg cells through the above binding partners or other unknown binding partner(s).

It has been reported that *in vitro* Th2/Treg differentiation is not impaired in the absence of autophagy-related gene Atg16l1 (5). Consistently, here we have found that the *in vitro* differentiation of naive RACK1-deficient CD4^+^ T cells was not impaired under Th1 polarization conditions. In addition, purified splenic T cells from *Gnb2l1*^F/F; CD4-Cre^ mice show no defect in IFN-γ expression after *in vitro* stimulation with Con A (Figure S6 in Supplementary Material). Thus, RACK1 does not directly affect IFN-γ expression in peripheral T cells. The reduced IFN-γ expression by RACK1-deficient T cells after Con A treatment may result from the improved microenvironment in the liver. As for NKT cells, we have found that CD3^+^NK1.1^+^CD1d-tetramer^−^ NKT cells accumulated in the liver of *Gnb2l1*^F/F; CD4-Cre^ mice despite that iNKT cells were almost absent. iNKT cells, but not other NKT cells, contribute essentially to Con A-induced liver injury (29, 30, 40). So NKT cells in the liver of *Gnb2l1*^F/F; CD4-Cre^ mice produce less IFN-γ. The origin and function of these accumulated CD3^+^NK1.1^+^CD1d-tetramer^−^ NKT cells in the liver of *Gnb2l1*^F/F; CD4-Cre^ mice remain to be explored.

## Ethics Statement

The study was approved by the ethics committee of the Institute of Basic Medical Sciences under the number AMMS376/2014. The use of animals was also approved by the Institute of Basic Medical Sciences.

## Author Contributions

JZ initiated and designed the experiments, and JG analyzed the data. GQ, JL, and QC performed most of the experiments. QW, ZJ, YP, and MZ were involved in FACS analysis. JW assisted in immunobltting analysis. GQ and JL wrote the initial draft of the manuscript, and JZ finalized the manuscript.

## Conflict of Interest Statement

The authors declare that the research was con-ducted in the absence of any commercial or financial relationships that could be construed as a potential conflict of interest.
