# Supplementary material for: Impaired Autophagy and Defective T Cell Homeostasis in Mice with T Cell-Specific Deletion of Receptor for Activated C Kinase 1
Source: Front Immunol. 2017 May 18;8:575. doi: 10.3389/fimmu.2017.00575 (PMC5435764; doi:10.3389/fimmu.2017.00575)
Supplement: Supplementary file 1 [file data_sheet_1.pdf]

Supplemental Material

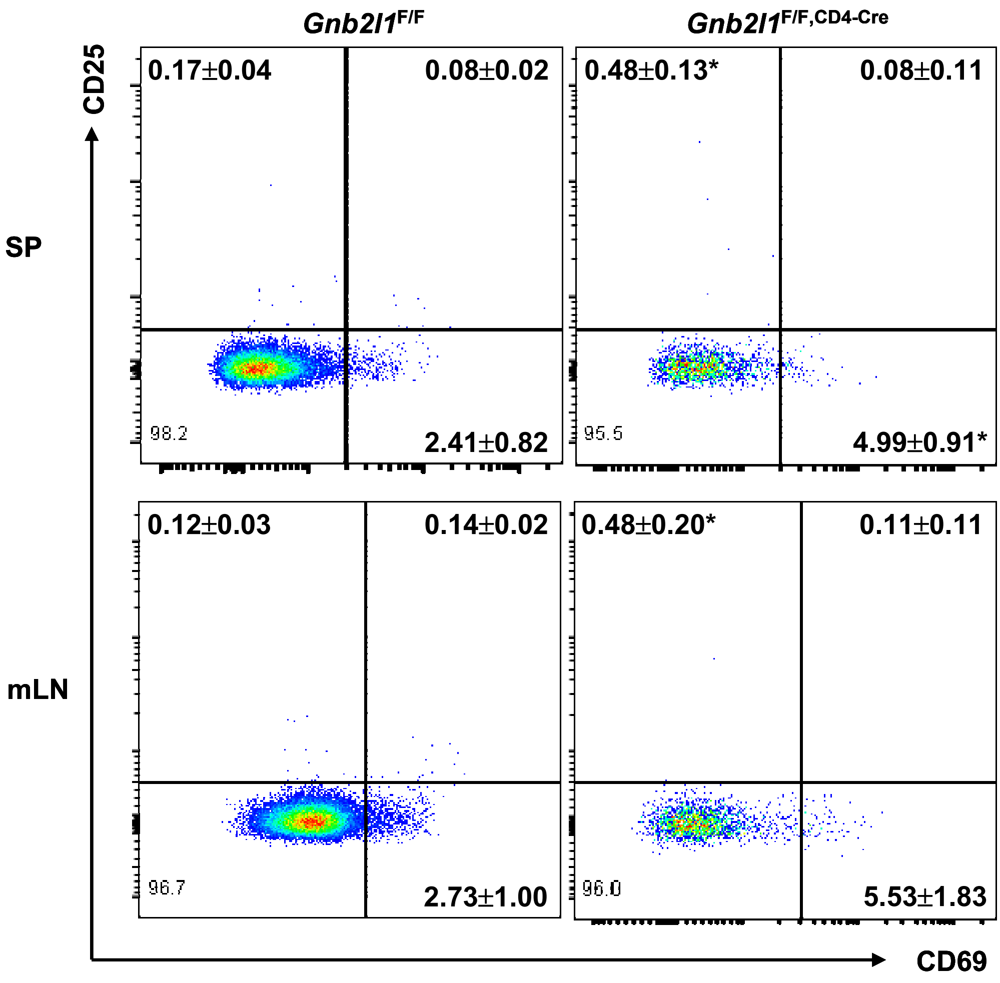

FIGURE S1 | Analysis of the expression profile of CD25 and CD69 in RACK1-sufficient and -deficient peripheral CD8+ T cells. Flow cytometric analysis of the expression of CD25 and CD69 in peripheral CD8+ T cells of 6- to 8-week-old *Gnb2l1*$^{F/F;\ CD4\text{-}Cre}$ mice, as compared to littermate controls. Representative plots and the mean percentages ± SD are shown (n=4). *$P < 0.05$ **$P < 0.01$.

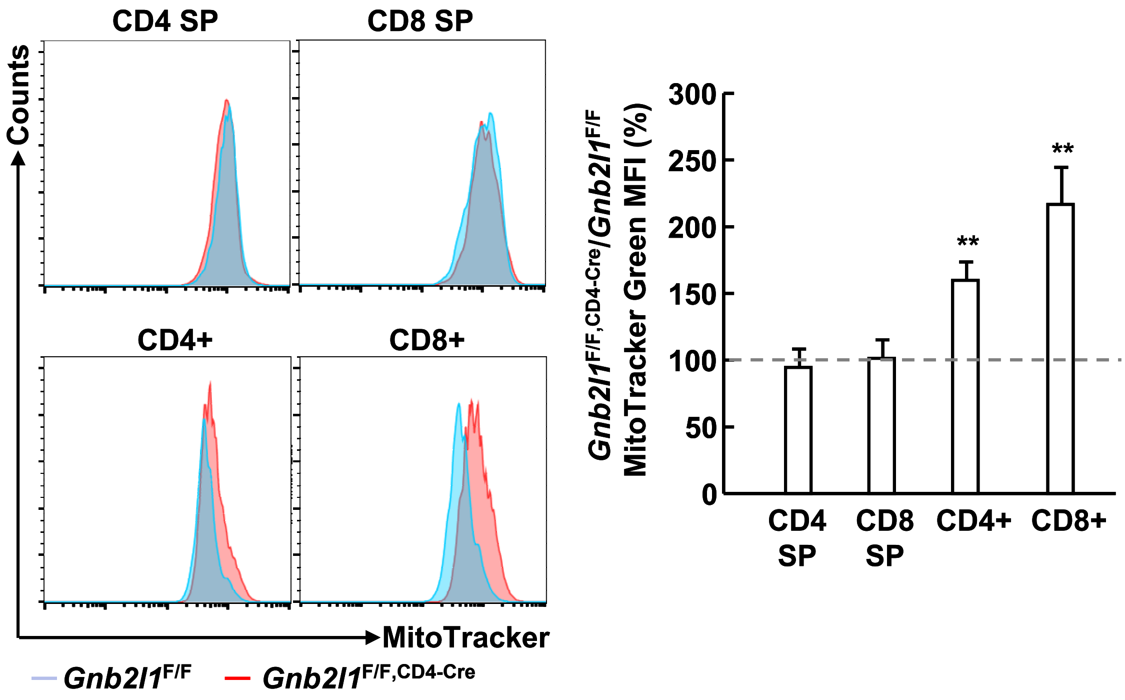

FIGURE S2 | Analysis of mitochondrial content in T cell subsets isolated from *Gnb2l1*$^{F/F; CD4\text{-}Cre}$ mice and littermate controls. Single-cell suspensions of thymi and spleens from 6- to 8-week-old *Gnb2l1*$^{F/F; CD4\text{-}Cre}$ mice and littermate controls were incubated with MitoTracker Green. *Left*, Representative plots of gated subsets. *Right* the mean percentages ± SD of MitoTracker Green mean fluorescent intensity (MFI) in gated RACK1-deficient vs. RACK1-sufficient subsets (n=4). Data shown in this figure are representative of at least three independent experiments. *$P < 0.05$ **$P < 0.01$.

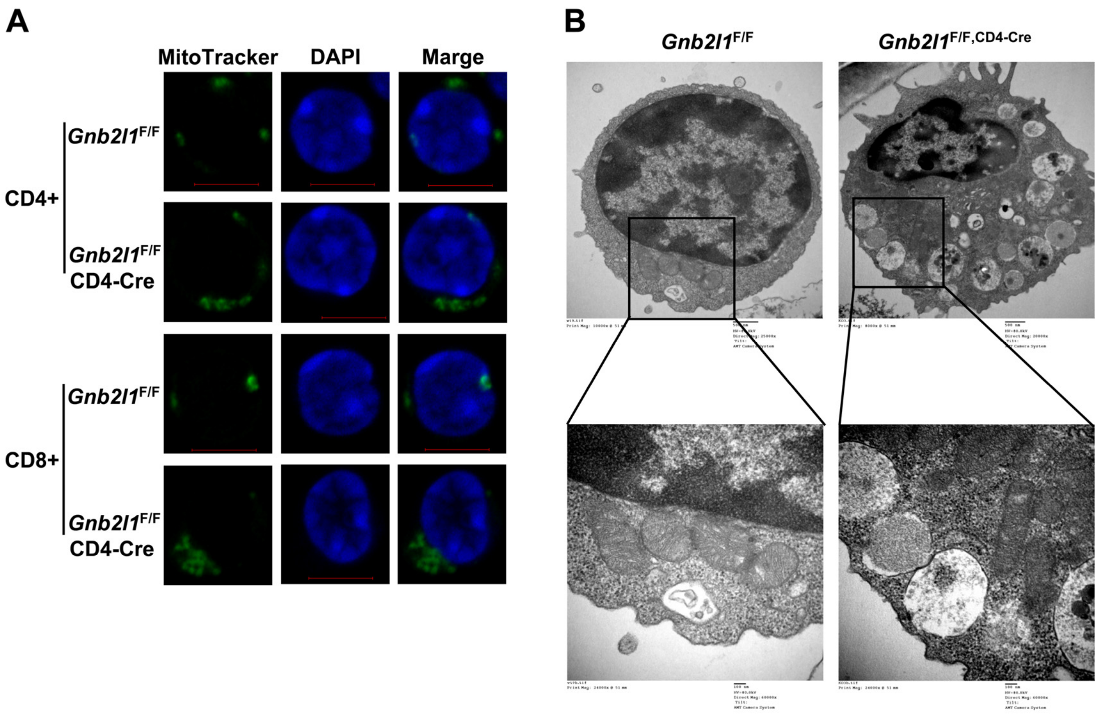

FIGURE S3 | Analysis of mitochondrial morphology in RACK1-sufficient and -deficient peripheral T cells (**A**) Peripheral T cells isolated from 6- to 8-week-old *Gnb2l1*$^{F/F;\ CD4\text{-}Cre}$ mice and littermate controls were incubated with 100 nM MitoTracker Green for 30 min at 37°C, followed by wash and fixation with 1% (w/v) paraformaldehyde. After incubation with 1 μg/ml 4′,6-diamidine-2-phenylindole (DAPI) for 5 min, the cells were subjected to confocal microscopy. Representative images are shown (Scale bar: 5 um) (**B**) CD8+ cells were purified from 6- to 8-week-old *Gnb2l1*$^{F/F;}$ $_{CD4\text{-}Cre}$ mice and littermate controls and subjected to electron microscopy. Representative images are shown. The lower panels (Scale bar: 100 nm) are detailed images of the upper panels (Scale bar: 500 nm).

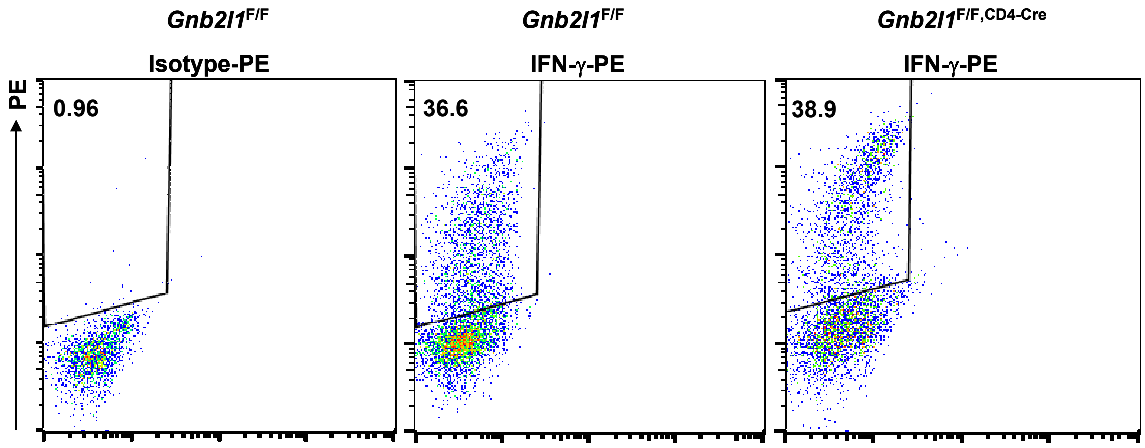

FIGURE S4 | Analysis of *in vitro* Th1 differentiation of RACK1-sufficient and -deficient naïve CD4+ T cells. Naïve CD4+ T cells purified from 6- to 8-week-old *Gnb2l1*$^{F/F;\ CD4\text{-}Cre}$ mice and littermate controls were stimulated with Dynabeads mouse CD3/CD28 T cell expanders (Invitrogen) 96-well plates at the density of 2.5×10^4 cells/well. 10 μg/ml anti-murine IL-4 antibody (eBioscience) and 10 ng/ml recombinant murine IL-12 (R&D Systems) was included. 5 days after T cell differentiation, cells were stimulated for 4 h with PMA (Sigma) and ionomycin (Sigma) in the presence of BFA (eBioscience), followed by flow-cytometric analysis of intracellular IFN-γ. Representative data of two independent experiments with similar results are shown.

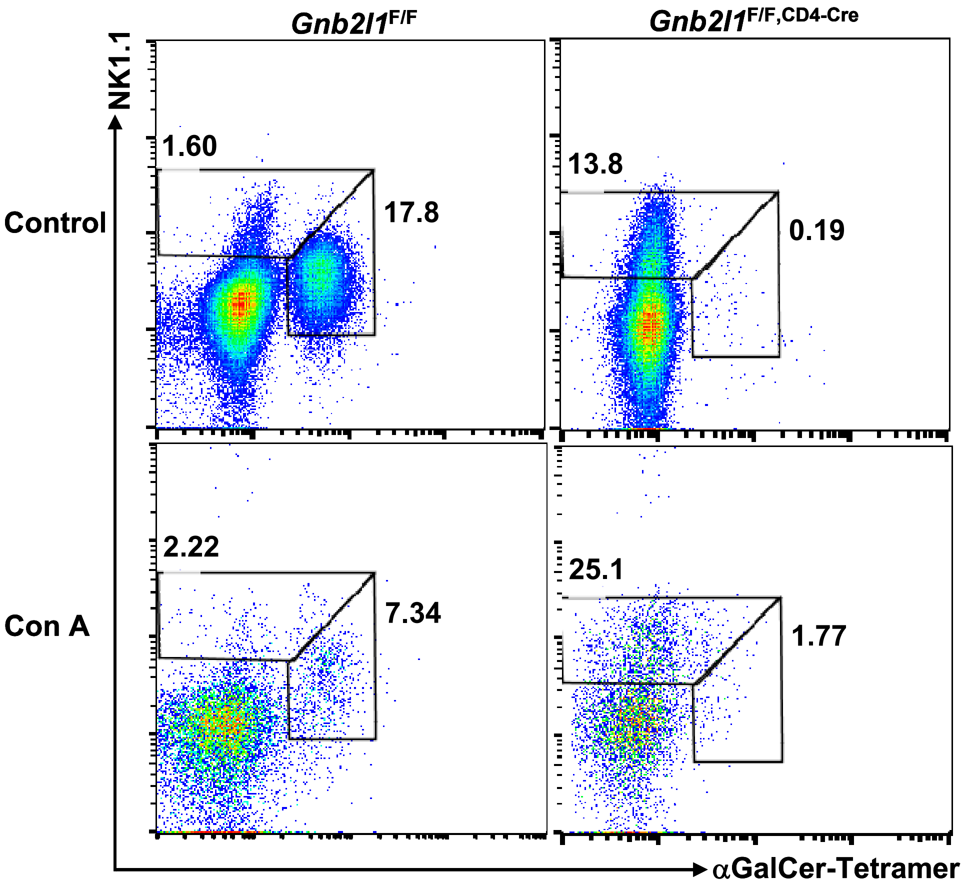

FIGURE S5 | Analysis of the relationship between CD3+NK1.1+ subset and CD3+CD1d-tetramer+ subset in the liver of *Gnb2l1*$^{F/F;\ CD4\text{-}Cre}$ mice and littermate controls. Liver mononuclear cells isolated from 6- to 8-week-old *Gnb2l1*$^{F/F;\ CD4\text{-}Cre}$ mice and littermate controls were stained with fluorescent-dye-labeled antibodies against CD3, NK1.1, and CD19 and αGalCer-loaded CD1d-tetramer, followed by flow cytometric analysis. CD19-negative and CD3-positive live cells were gated. Representative data of two independent experiments with similar results are shown.

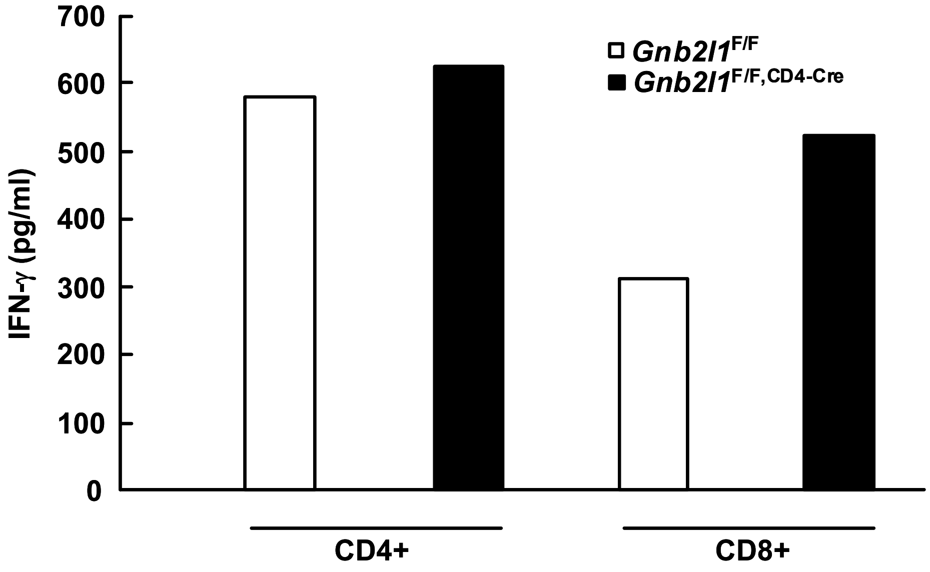

FIGURE S6 | Analysis of IFN-γ secretion by purified RACK1-sufficient and –deficient splenic T cells after Con A stimulation. Splenic T cells purified from 6- to 8-week-old *Gnb2l1*$^{F/F;\ CD4\text{-}Cre}$ mice and littermate controls were stimulated with Con A (5 μg/ml). 48 h later, the supernatants were harvested and subjected to ELISA to determine IFN-γ concentration according to the manufacturer's protocol. The ELISA kit for IFN-γ was purchased from R&D Systems. The secretion of IFN-γ by splenic T cells was calculated according to the cell number. Representative data of two independent experiments with similar results are shown.
